# Investigating the diagnostic and prognostic significance of genes related to fatty acid metabolism in hepatocellular carcinoma

**DOI:** 10.1186/s12876-024-03495-2

**Published:** 2024-11-15

**Authors:** Sha-Sha Zhao, Rong-Rong Bai, Bao-Hua Zhang, Xiao-Rui Sun, Nan Huang, Yan Chen, Zi-Jiu Sun, Li-Mei Sun, Yue Zhang, Zhong-Qi Cui

**Affiliations:** 1 Department of Clinical Laboratory, Shanghai Tenth People’s Hospital, Tongji University School of Medicine, Shanghai, 200092 China; 2 Department of Clinical Laboratory, Clinical Medicine Scientific and Technical Innovation Park, Shanghai Tenth People’s Hospital, Shanghai, 200435 China; 3 Suzhou Medical College of Soochow University, Suzhou, PR China; 4 Shanghai YK Pao School, Shanghai, 201620 China

**Keywords:** Fatty acid metabolism-related genes, Weighted gene co-expression network analysis, Diagnosis, Prognosis, Biomarker, *SLC22A1*, Hepatocellular carcinoma

## Abstract

**Background:**

Hepatocellular carcinoma (HCC) is one of the most prevalent and lethal cancers worldwide, with death rates increasing by approximately 2–3% per year. The high mortality and poor prognosis of HCC are primarily due to inaccurate early diagnosis and lack of monitoring when liver transplantation is not feasible. Fatty acid (FA) metabolism is a critical metabolic pathway that provides energy and signaling factors in cancer, particularly in HCC, and promotes malignancy. Therefore, it is essential to explore specific FA metabolism-related diagnostic and prognostic signatures that can enable the effective early diagnosis and monitoring of HCC.

**Methods:**

In this study, we used genes associated with FA metabolism pathway and weighted gene co-expression network analysis (WGCNA) to establish a gene co-expression network and identify hub genes related to HCC (disease WGCNA) and FA clusters (cluster WGCNA). A diagnostic model was constructed using data downloaded from the Gene Expression Omnibus database (GSE25097), and a prognostic model was established using The Cancer Genome Atlas cohort, in which Univariate Cox regression analysis, multivariate Cox risk model, and LASSO Cox regression analysis were applied. The immune infiltration of HCC cells was evaluated using CIBERSORT. The function of the key SLC22A1 gene was experimentally verified in vitro and in vivo.

**Results:**

Twelve overlapping genes (*CPEB3*, *ASPDH*, *DEPDC7*, *ETFDH*, *UGT2B7*, *GYS2*, *F11*, *ANXA10*, *CYP2C8*, *GLYATL1*, *C6*, and *SLC22A1*) from disease and cluster WGCNA were identified as key genes and used in the construction of the diagnostic and prognostic models. The RF model had the highest area under the ROC curve (AUC) of 0.994 was identified as the most effective for distinguishing patients with HCC with different features. The top five important genes (*C6*, *UGT2B7*, *SLC22A1*, *F11*, and *CYP2C8*) from the RF model were selected as diagnostic genes for further analysis (ROC curves: AUC value = 0.986, 95% confidence interval [95% CI] = 0.967–0.999). Moreover, a risk score formula consisting of four genes (GYS2, F11, ANXA10 and SLC22A1) was established and its independent prognostic ability was further demonstrated (univariate Cox regression analysis: hazard ratio [HR] = 3.664%, 95% CI = 2.033–6.605, *P* < 0.001; multivariate Cox regression analysis: HR = 2.801%, 95% CI = 1.553–5.049, *P* < 0.001). Additionally, in vitro and in vivo experiments demonstrated that SLC22A1 inhibits HCC tumor development, suggesting it may be a potential therapeutic target for HCC.

**Conclusions:**

These findings indicate a considerable value of specific FA metabolism-related genes in the diagnostic and prognostic evaluation of HCC, which provide novel insights into the disease’s management, as well as has potential implications for personalized treatment strategies. However, further investigation of the effects of these model genes on HCC is required.

**Supplementary Information:**

The online version contains supplementary material available at 10.1186/s12876-024-03495-2.

## Background

Primary liver cancer is the third deadliest cancer worldwide, with a mortality rate that is nearly equivalent to the annual incidence rate. This suggests that the majority of patients had a survival time of less than a year. In 2022, there were about 800,000 reported deaths attributable to this disease [1, 2]. Hepatocellular carcinoma (HCC) accounts for approximately 70–85% of the total liver cancer burden. Although early detection and surgical resection can lead to five-year survival rates of more than 70% [3], most patients in China are diagnosed with advanced disease, resulting in 5-year survival rates of less than 12.5% [4]. The high mortality and poor prognosis of HCC can be primarily attributed to the inaccuracy of early diagnosis and lack of monitoring. Although ultrasonography and alpha-fetoprotein serological assessment have been the main diagnostic approaches for HCC, their diagnostic accuracy and specificity, particularly in the early stages of the disease, have been unsatisfactory [5, 6].

HCC is mainly associated with alcohol abuse, hepatitis C virus, hepatitis B virus [7], obesity [8]and type 2 diabetes [9]. However, recent research has indicated that non-alcoholic fatty liver disease may be a potential cause in 13–38.2% of patients with HCC who do not have any viral or alcohol-related factors [10]. To overcome nutritional deficiencies and support rapid proliferation, cancer cells undergo metabolic reprogramming, which involves alterations in amino acid and glucose metabolisms. Among these, the “Warburg effect” is the most well-known phenomenon [11–13]. Lipids are crucial components of the plasma membrane and play key roles in cellular functions. Reprogramming of fatty acids (FAs) has recently emerged as an important factor contributing to tumor development, metastasis, and poor prognosis [14–18]. Studies have demonstrated dysregulated FA metabolism in HCC, and targeting associated signaling pathways may have potential therapeutic value [19–21]. Moreover, genes involved in FA metabolism may serve as important diagnostic and prognostic markers, although their precise implications remain largely unknown and merit further investigation.

Therefore, in this study, we aimed to explore specific FA-related genes as diagnostic and prognostic signatures for the effective early detection and monitoring of HCC. We comprehensively analyzed the diagnostic and prognostic values of FA-related genes in patients with HCC using online integrated bioinformatics tools. Our findings suggest that FA-related genes have potential as diagnostic and prognostic biomarkers for HCC, offering valuable insights into predicting HCC and enhancing treatment efficacy through personalized approaches.

## Methods

### Data collection and acquisition of FA-related genes

The microarray data GSE25097, including the series matrix and platform files, were obtained from the GEO database (http://www.ncbi.nlm.nih.gov/geo/). The GSE25097 dataset was extracted from the GPL10687 platform (Human RSTA Affymetrix 1.0) and consisted of 268 HCC tumor and 243 adjacent non-tumor samples. After downloading the data, the datasets were annotated using the “Perl” programming language. Subsequently, the annotated datasets were normalized using the R packages “BiocManager” and “limma”.

We obtained a list of genes associated with FA metabolism pathway (FA-related genes) from the publicly available MSigDB database [22], specifically from the “HALLMARK FATTY ACID METABOLISM” category. The expression levels of the FA-related genes were extracted from the normalized GEO dataset and converted into log2 values. These log2-transformed expression levels were then used to identify FA-related differentially expressed genes (FAs-DEGs) using the “limma” R package. The selection criteria for FAs-DEGs were log (fold-change) values ≥ 0.5 and false discovery rate (FDR) < 0.05.

Additionally, RNA sequencing and clinical data (including gdc and cart files) for Liver Hepatocellular Carcinoma (LIHC) from TCGA database (https://gdc.cancer.gov/), consisting of 50 normal and 374 tumor samples, were downloaded. The downloaded files were converted into Gene Symbols using the “Perl” programming language, and relevant clinical information was extracted.

### FA clusters in HCC identification

To identify the expression patterns related to FAs in HCC, Wilcox.test was performed to detect DEGs associated with FAs, using a significance level of *P* < 0.05. Subsequently, the consensus clustering algorithm (Consensus Cluster Plus) was used to categorize the 268 HCC samples into distinct clusters based on the expression profiles of FA-DEGs.

### Co-expression network establishment and hub module screening

We used the “WGCNA” R package to construct co-expression modules [23]. To ensure accurate results, we first screened the top 25% of the genes with the highest variance and constructed FAs clusters for two subsequent WGCNA analyses. The optimal soft power of 4 and a scale-free R^2^ of 0.9 were selected to establish a weighted adjacency matrix, which was then converted into a topological overlap matrix (TOM). For module identification, we used a hierarchical clustering tree algorithm with the minimum module size set to 100 using the TOM dissimilarity measure (1-TOM).

Each module was assigned a different color. To identify and explore the significant modules, we calculated the correlations between the analyzed clinical phenotypes and modules using Pearson’s tests. A distinct module with *P* < 0.05 was considered to be significantly correlated, while the module with the highest correlation coefficient was labelled the “hub” module. Hub genes within each module were identified based on geneSigFilter > 0.5 and moduleSigFilter > 0.7.

### Diagnostic model construction and assessment using machine learning models

To construct a predictive model, we first utilized the R package “VennDiagram” to identify the overlapping hub genes obtained from the two WGCNA analyses, which were then defined as key genes. Subsequently, we established four machine learning models based on these key genes using the “caret” R package. The random forest (RF) model, a method based on the regression tree technique, utilizes bootstrap aggregation and randomization of predictors to achieve a high degree of predictive accuracy [24]. The support vector machine (SVM) algorithm, a powerful technique for both regression and classification, enables the identification of negative and positive instances by locating the maxima of exact evidence [25]. The generalized linear model (GLM), an extension of multiple linear regression models, is flexible in evaluating the relationship between the dependent features of a normal distribution and classification or continuous independent features [26]. The extreme gradient boosting (XGB) technique, allows for a precise comparison between the classification error and model complexity. Following the establishment of these machine learning models, we applied the “DALEX” and “pROC” R packages to estimate the residual and diagnostic value of the models.

Next, we adopted Logistic Regression Model and nomogram to evaluate the incidence of HCC clusters using the “rms” R package. The model assigns corresponding points for single factors and total points for indicators of combinations of all factors. The predictive power of the nomogram model was assessed using calibration and decision curve analysis (DCA).

We further validated the predictive power of the model using the TCGA dataset with the “pROC” R package. Additionally, we analyzed the differential expression levels of the model genes between normal and HCC samples in both TCGA and GSE25097 datasets.

### Immune cell infiltration evaluation and correlation between RF model genes and infiltrated immune cell analysis

To evaluate the relative infiltration levels of 22 immune cell types in each sample, we utilized the CIBERSORT algorithm (https://cibersort.stanford.edu/) and the LM22 signature matrix for analysis of the normalized GSE25097 dataset. Additionally, we employed the R packages “limma,” “reshape2,” “tidyverse,” and “ggplot2” to further assess the association between RF model genes and the infiltrated immune cells. Statistical significance was set at *P* < 0.05.

### FA-related genes prognostic model establishment

First, key genes used to construct the diagnostic model were employed to establish a prognostic model. Univariate Cox regression analysis was conducted on the key genes, and TCGA data were processed to identify potential prognostic indicators. Subsequently, these indicators were incorporated into a multivariate Cox risk model. In constructing the multivariate Cox risk model, we utilized the R package “glmnet” and performed LASSO Cox regression analysis to generate a risk score formula. Finally, four genes with significant prognostic potential were included in the risk score signature, calculated as follows:

$$\eqalign{ Risk score & = \left({Coefficien{t_{gene1}}*Ex{p_{gene1}}} \right) \cr & + \left({Coefficien{t_{gene2}}*Ex{p_{gene2}}} \right) \cr & + \left({Coefficien{t_{gene3}}*Ex{p_{gene3}}} \right) \cr & + \left({Coefficien{t_{gene4}}*Ex{p_{gene4}}} \right) \cr}$$

377 patients with HCC of different stages of disease in TCGA dataset were classified into low- and high-risk groups based on their median risk score. The Kaplan–Meier method and log-rank test were used to compare the overall survival (OS) rates between subgroups. We used the R package “Survival” to construct a receiver operating characteristic (ROC) curve and evaluate the predictive power of the established model. Furthermore, the R package “Survival” was utilized to assess the independent prognostic value and association with clinical characteristics of our prognostic model. Additionally, the validity of the prognostic model was further performed in GSE14520 dataset.

### Immunohistochemistry (IHC) staining analysis and qRT-PCR

IHC and qRT-PCR analyses were performed on residual HCC tissues after routine pathological examination obtained from 47 patients who underwent surgery at the Shanghai Tenth People’s Hospital between May 2020 and July 2023. In addition, 47 para-carcinoma tissue samples were collected from the same patients as the controls. The age range of the tissue donors was 40–65 years. This study was approved by the Ethics Committee of the Shanghai Tenth People’s Hospital (Approval Number: 21K196).

For IHC analysis, tissue specimens were fixed in 4% paraformaldehyde at 25 ℃, followed by detection using a previously outlined method [27].

Total RNA was extracted, and qRT-PCR was performed to determine the expression of *SLC22A1*. The primer sequences used for *SLC22A1* were as follows: forward primer: 5ʹ-ACGGTGGCGATCATGTACC-3ʹ and reverse primer: 5ʹ-CCCATTCTTTTGAGCGATGTGG-3ʹ. *GAPDH* served as an endogenous control.

### Cell culture and overexpression vector transfection

SNU449 and SNU398 cell lines were obtained from the American Type Culture Collection in Maryland, USA. Cells were cultured in RPMI-1640 medium (Gibco, USA) supplemented with 10% fetal bovine serum (ExCell Bio, Uruguay) and 1% antibiotics (penicillin-streptomycin; Gibco). The cells were maintained at 37 °C in a 5% CO_2_ atmosphere.

To establish the overexpression vectors, the coding sequences of the target genes were amplified and subcloned into the pcDNA 3.1 vector (Invitrogen, USA). An empty vector was used as a negative control. Transfection of the overexpression plasmids into cells was performed using Lipofectamine 2000 (Invitrogen) and serum-free Opti-MEM (Gibco) according to the manufacturer’s instructions.

### Western blotting

Whole cell lysates were prepared using RIPA buffer (0.1 g/mL) (Beyotime, China) supplemented with 1% (v/v) protease and phosphatase inhibitor cocktail (Thermo Fisher Scientific, Waltham, MA, USA) and 1% (v/v) phenylmethanesulfonyl fluoride. Before 10% sodium dodecyl sulphate-polyacrylamide gel electrophoresis, BCA protein quantification was performed to ensure equal protein loading. Nitrocellulose membranes (Millipore EMD, Billerica, MA, USA) were used for protein transfer and blocked with 5% (w/v) skim milk in phosphate-buffered saline-Tris at RT for 1 h. The blots were cut prior to hybridisation with antibodies and then incubated overnight at 4 °C with primary antibodies, followed by incubation with the secondary antibodies at 25 °C for 1 h. Finally, the blots were visualized using the Image Studio Ver 5.2 system (Odyssey CLX, LICOR, USA).

### Cell counting kit (CCK)-8, EDU and clone formation assays

For the CCK-8 assays, 1 × 10^3^ cells in 100 µL of medium were added to each well of a 96-well plate. For EDU assays, 2 × 10^4^ cells per well were plated in a 96-well plate. CCK-8 (Vazyme Biotech, China) and EdU Cell Proliferation Kit with Alexa Fluor 555 (Beyotime) were used to assess the proliferative ability of the HCC cells, following the manufacturer’s instructions.

For the clone formation assays, 1 × 10^3^ cells per well were seeded in a 12-well plate and incubated in a 37 °C, 5% CO^2^ incubator for 7 days. The cells were fixed with 4% paraformaldehyde and stained with crystal violet.

### Transwell assays

For the transwell assays, 5 × 104 cells in 200 µL of serum-free medium were added to the upper chamber of a transwell insert, while 500 µL of RPMI-1640 containing 20% FBS was placed in the bottom chamber. After incubation at 37 °C with 5% CO_2_ for 48 h, the migrated cells in the chambers were fixed with 4% paraformaldehyde, stained with crystal violet, and photographed under a microscope.

### Measurements of cell death

Cell death was measured using a previously published protocol [28]. Briefly, the treated cells were stained with propidium iodide (1:1000; Invitrogen) in the absence of light for 5 min at 25 °C and subsequently analyzed using flow cytometry.

### Animal studies

Animal studies were conducted in compliance with the ethical permission granted by the Institutional Animal Care and Use Committee of the Shanghai Tenth People’s Hospital, Shanghai, China (Approval Number: SHDSYY-2021-4709). For the tumor formation assay, a total of ten male nude mice [29, 30], aged 5 weeks, were procured from Shanghai Jihui experimental animal feeding Co., LTD. Mice were housed in SPF animal houses with a maximum of five mice per cage, which were subcutaneously injected with 5 × 10^6^ cells suspended in PBS, either on the left or right flank. Tumor volume was assessed every three days and calculated using the formula:

Volume (mm^3^) = 0.5 × Length (mm) × Width^2^ (mm^2^).

After 27 days, the mice were euthanized and the subcutaneous tumors were excised, weighed, and photographed. Cervical vertebrae dislocation after carbon dioxide anesthesia was employed as the euthanasia/sacrifice methods, which can reduce the pain of mice to the greatest extent.

### Statistical analysis

Data were analyzed using GraphPad Prism 7 and R version 4.2.2. Experimental results were presented as mean ± standard deviation and the Wilcoxon signed-rank and Kruskal-Wallis tests were used for between-group comparisons. Statistical significance was established as by *P* < 0.05 (*P < 0.05, **P < 0.01, ***P < 0.001).

## Results

### Data processing protocol and mRNA expression profiles

**Fig. 1:**
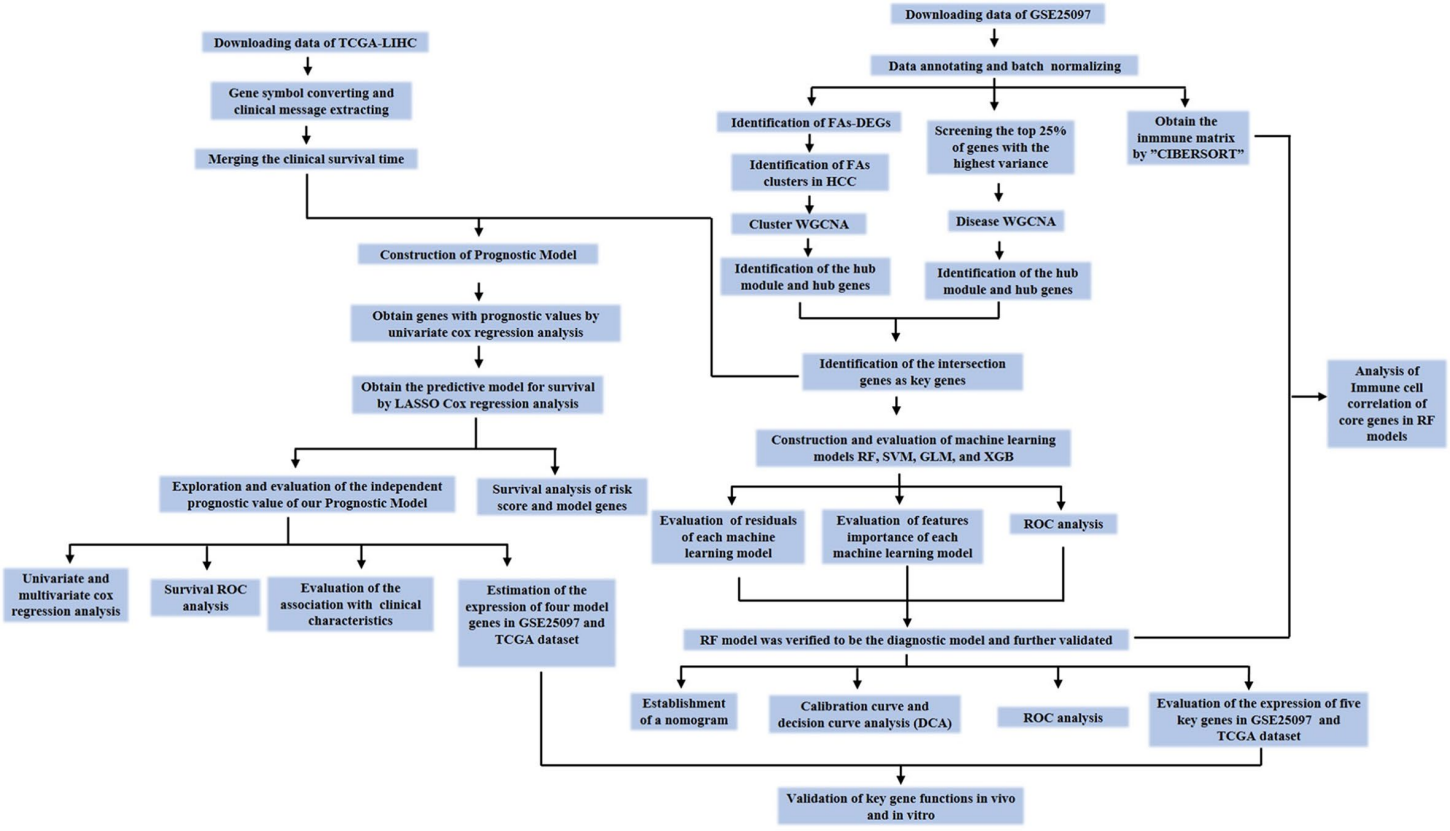
Diagnostic and prognostic significance workflow of FA-related genes in hepatocellular carcinoma (HCC) using WGCNA algorithms. This analysis involves leveraging data obtained from both the GEO and TCGA databases

The diagnostic and prognostic significance of the FA-related genes in HCC was investigated as illustrated in Fig. 1. The mRNA expression profiles from the GEO and TCGA-LIHC dataset were included in the analysis.

### Identification of FA clusters in HCC

**Fig. 2:**
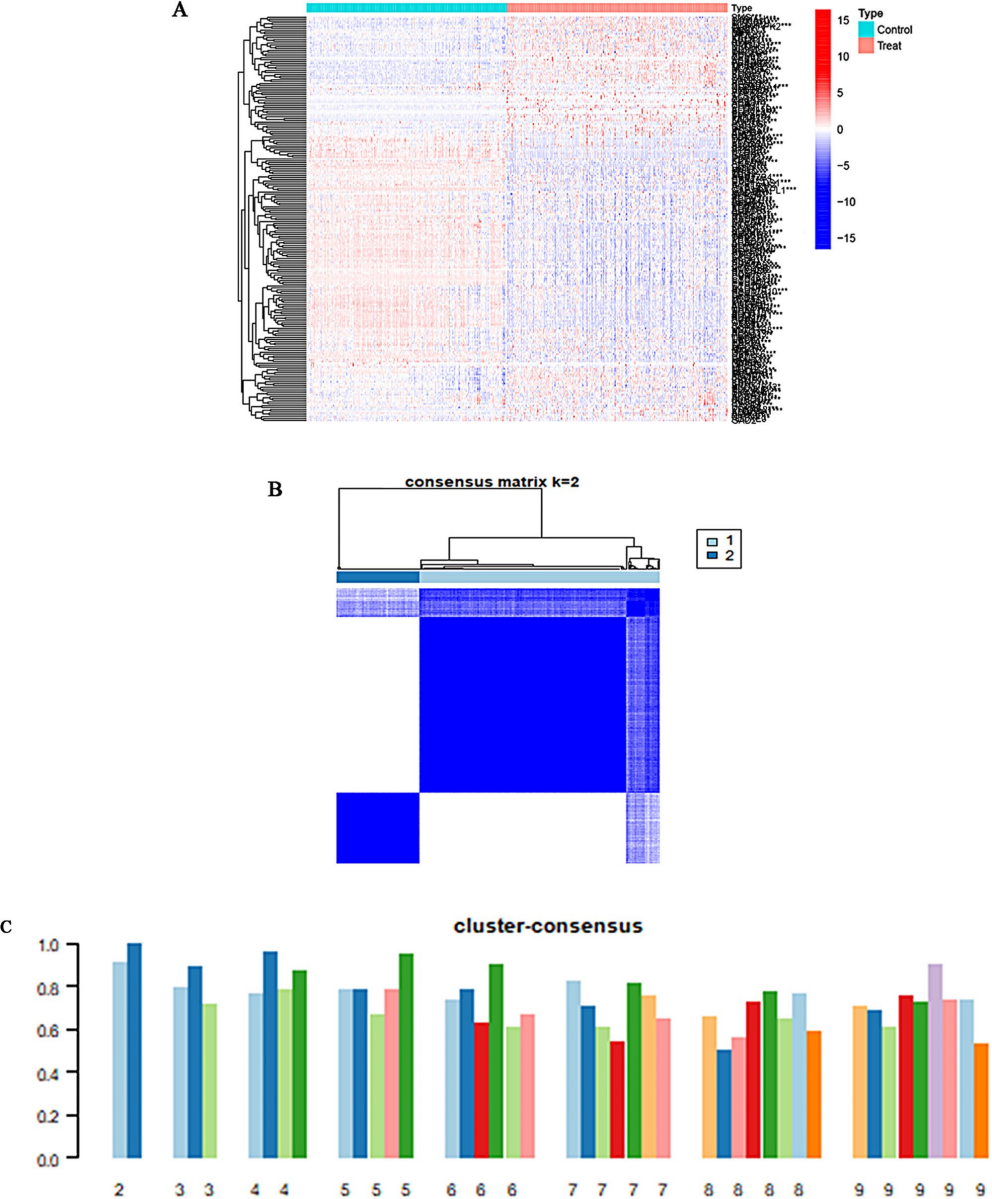
Identification of FAs clusters in HCC. (**A**) The heatmap displaying the expression levels of 220 FA-DEGs in normal and tumour tissue of HCC. Blue and red colours indicate low and high expression of genes, respectively. (**B**) Two FAs clusters (k = 2) were identified in HCC using a consensus clustering algorithm. (**C**) The consistency score of each subtype. The cluster-consensus was highest when the k value was set to two (k = 2). DEG, differential expressed gene; FA, fatty acid; HCC, hepatocellular carcinoma

To investigate the expression patterns of FA-related genes in HCC, we initially identified 220 FA-DEGs using the Wilcoxon test (Fig. 2A). Subsequently, we performed consensus clustering on the expression profiles of 220 FA-DEGs in 268 HCC samples. Consistently, the highest cluster consensus was observed when the clustering parameter k was set to two (k = 2), with a consistency score above 0.8 for each subtype (Fig. 2B, C).

### Establishment of the co-expression network and screening of hub modules

**Fig. 3:**
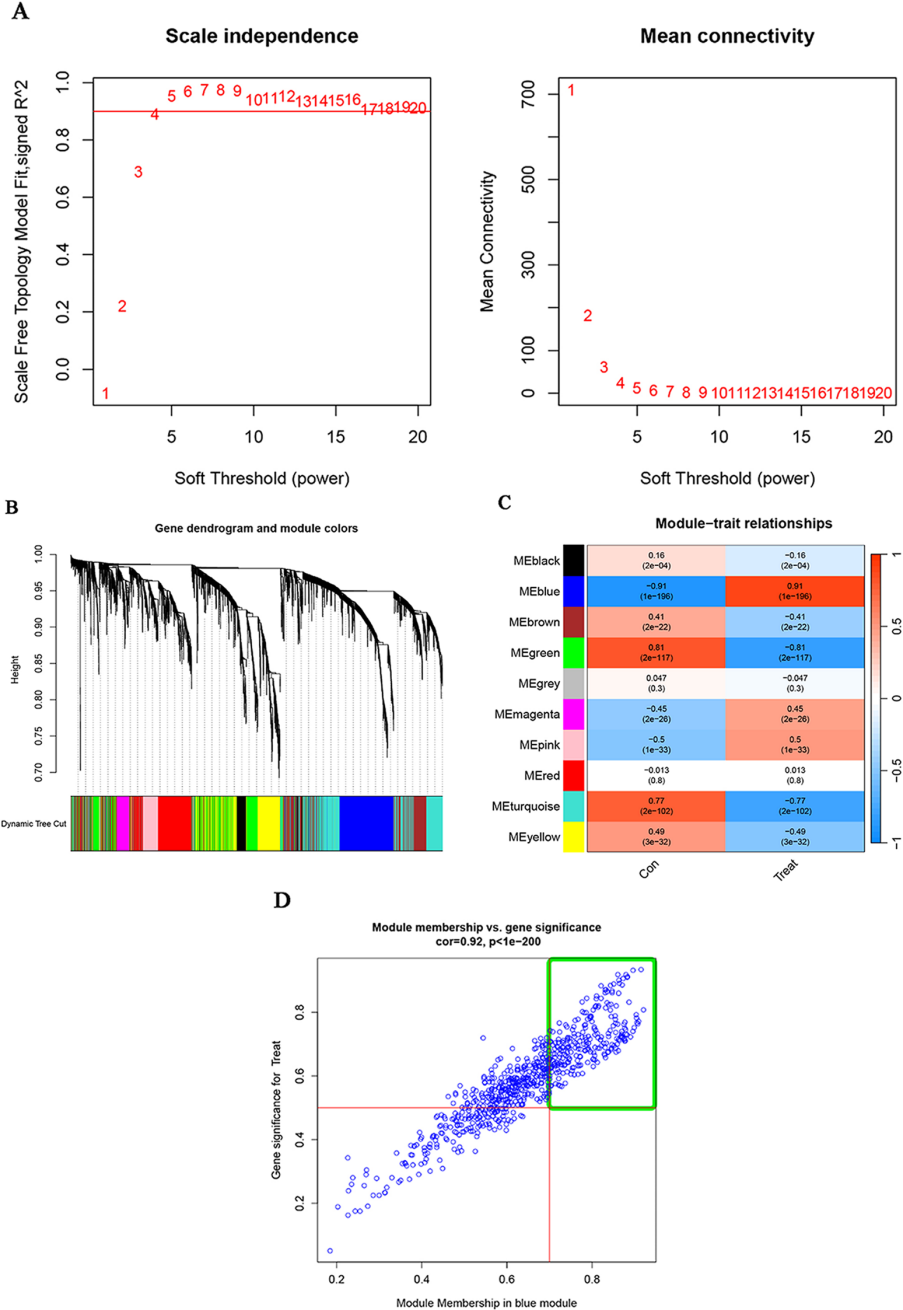
Co-expression network construction. The top 25% of genes with the highest variance in HCC were used. (**A**) The estimation of the soft threshold power was set to β = 4. (**B**) Co-expression modules were visualized as a cluster tree dendrogram, represented in various colours. (**C**) The relationships between consensus module eigengenes and HCC were examined. The rows in the figure correspond to consensus modules, while the columns represent clinical status. The numbers within each module indicate the correlation coefficients between the respective module and clinical status, along with the p-values enclosed in parentheses. The modules are depicted in different colours, where red signifies a positive correlation, and green signifies a negative correlation. (**D**) Module membership within the blue modules and gene significance in relation to HCC were analysed. Each blue dot represents a gene. Genes with module membership values > 0.7 and gene significances > 0.5, contained within the green box, were identified as candidate hub genes. HCC, hepatocellular carcinoma

The top 25% of genes with the highest variance in GSE25097 were identified and used to construct a co-expression network using the WGCNA R package to screen for associated modules in healthy individuals and those with HCC. The optimal soft power was 4 when the scale-free R^2^ set at 0.9 (Fig. 3A). Then, a hierarchical clustering tree was constructed (Fig. 3B) and ten distinct co-expression gene modules were generated (Fig. 3C). These 10 modules were displayed in different colors, and the blue module, which showed the strongest relationship with HCC, was identified as the hub module (cor = 0.91, *P*<0.001). A total of 349 genes with module membership values > 0.7 and gene significance values > 0.5 contained in this hub module, were considered hub genes. Furthermore, a positive association was observed between module-related genes and the blue module (cor = 0.92, *P* < 0.001) (Fig. 3D).

**Fig. 4:**
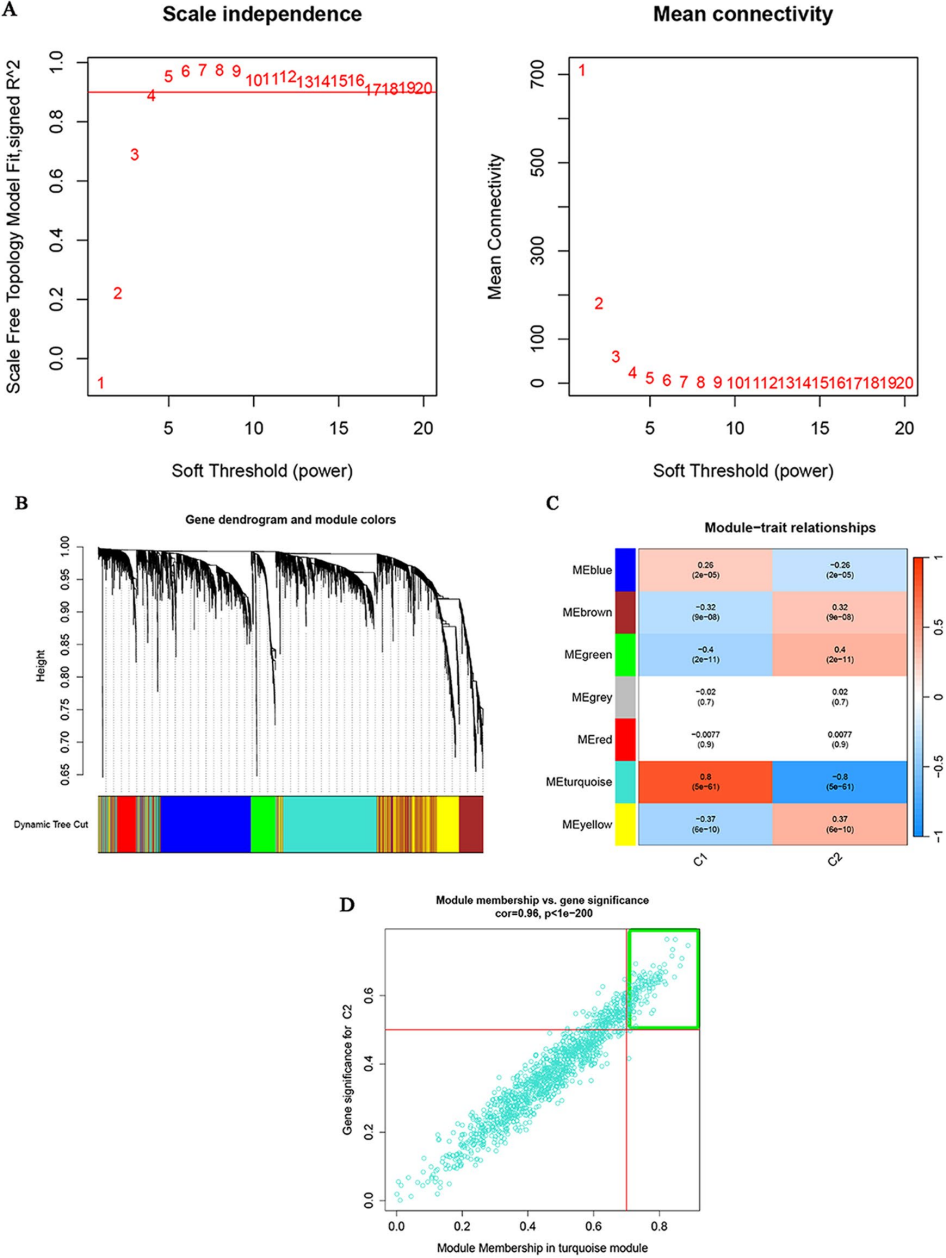
The co-expression network was constructed using FA clusters. (**A**) The soft threshold power was estimated to be β = 4. (**B**) Co-expression modules were visualized as a cluster tree dendrogram, distinguished by various colours. (**C**) The relationships between consensus module eigengenes and FA clusters were explored. The figure rows represent consensus modules, while the columns correspond to FAs clusters. The numbers within each module indicate the correlation coefficients between the respective module and clusters, along with the p-values enclosed in parentheses. The modules were visualized in different colours, where red signifies a positive correlation and green signifies a negative correlation. (**D**) Module membership within the turquoise modules and gene significance for clusters 2 were analysed. Each turquoise dot represents a gene. Genes with module membership values > 0.7 and gene significances > 0.5, located within the green box, were identified as candidate hub genes. FA, fatty acid

The WGCNA algorithm was used to analyze the key gene modules closely associated with FAs clusters, referred to as cluster WGCNA. A soft power of 4 and a scale-free R2 of 0.9 were determined to be the most suitable values for constructing a scale-independent and mean connectivity network (Fig. 4A, B). As a result, seven modules were generated as significant modules (Fig. 4C), and the hub module with the highest connectivity to HCC clusters was identified as the turquoise module (cor = 0.8, *P* < 0.001). This turquoise module consisted of 125 hub genes, which have module membership values > 0.7 and gene significance values > 0.5. Moreover, a significant relationship was observed between the genes in the turquoise module and the entire module (cor = 0.96, *P* < 0.001) (Fig. 4D).

### Construction and assessment of the diagnostic model

**Fig. 5:**
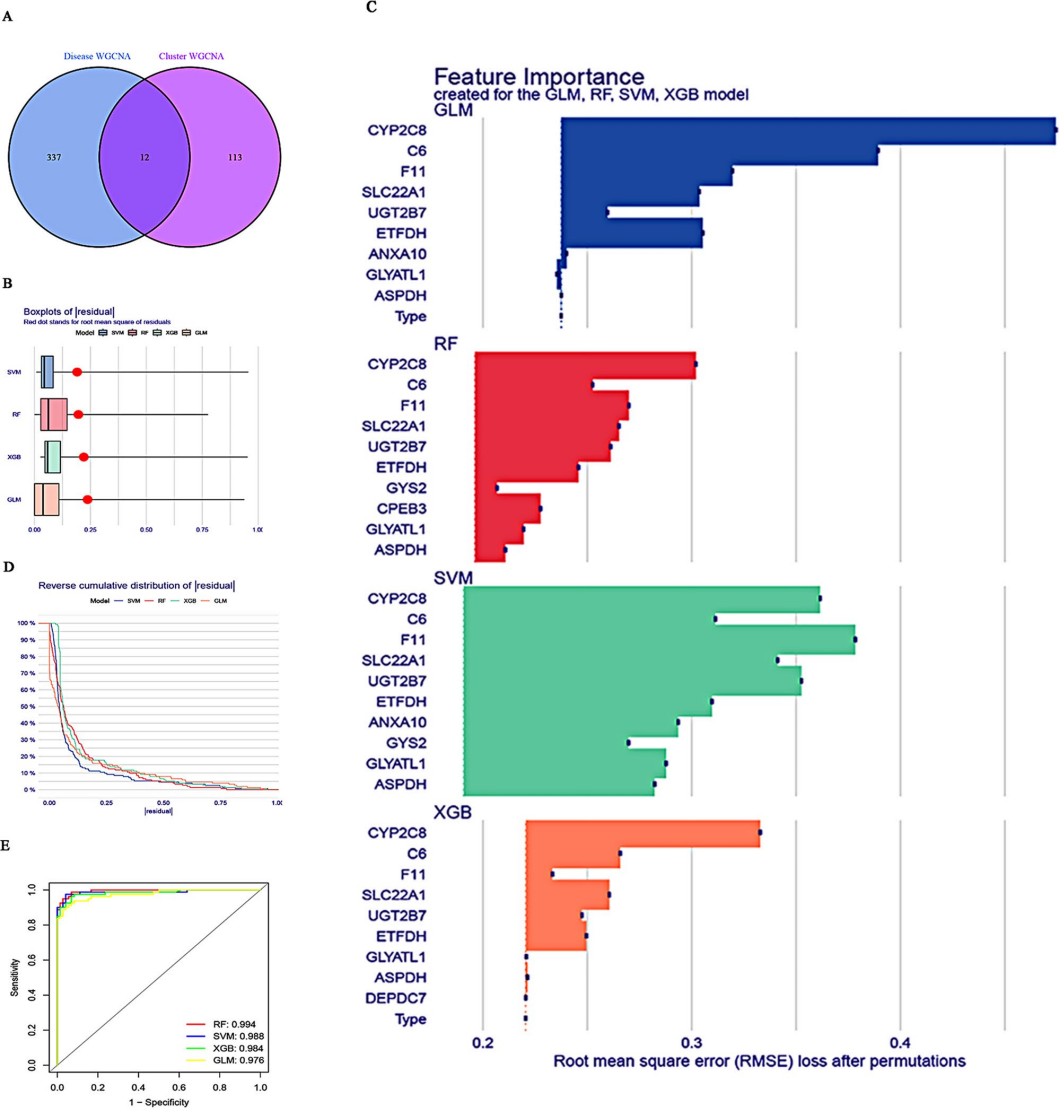
The machine learning models RF, SVM, GLM, and XGB were constructed and evaluated. (**A**) The intersections between genes related to hub modules, identified through disease WGCNA and cluster WGCNA, were investigated. (**B**) Boxplots of residuals were generated for each machine learning model, with red dots representing the root mean square of residuals (RMSE). (**C**) The feature importance of the RF, SVM, GLM, and XGB models were analysed. (**D**) The cumulative distribution of residuals was examined for each machine learning model. (**E**) ROC analysis of the four models, based on five-fold cross-validation in the GEO dataset, was conducted. GLM, generalized linear model; RF random forest; ROC, receiver operating characteristic; SVM, support vector machine; WGCNA, weighted gene co-expression network analysis; XGB, extreme gradient boosting

The diagnostic value of screened genes was further analyzed by identifying 12 overlapping genes (*CPEB3*, *ASPDH*, *DEPDC7*, *ETFDH*, *UGT2B7*, *GYS2*, *F11*, *ANXA10*, *CYP2C8*, *GLYATL1*, *C6*, and *SLC22A1*) among the hub genes identified in disease and cluster WGCNA using a Venn diagram (Fig. 5A). Next, four certified machine learning models (SVM, RF, XGB, and GLM) were established based on the expression profiles of the GSE25097 dataset and 12 potential key genes. Among these models, SVM and RF showed relatively low residual values (Fig. 5B, D). The top five features of each model were then permuted based on the root mean square error (Fig. 5C). Additionally, ROC curves based on five-fold cross-validation were calculated to estimate the diagnostic value of the four models in the testing set. The RF model had the highest area under the ROC curve (AUC) of 0.994, followed by the SVM (AUC = 0.988), GLM (AUC = 0.976), and XGB (AUC = 0.984) models (Fig. 5E). Based on these findings, the RF model was identified as the most effective for distinguishing patients with HCC with different features. The top five important genes (*C6*, *UGT2B7*, *SLC22A1*, *F11*, and *CYP2C8*) from the RF model were selected as diagnostic genes for further analysis.

**Fig. 6:**
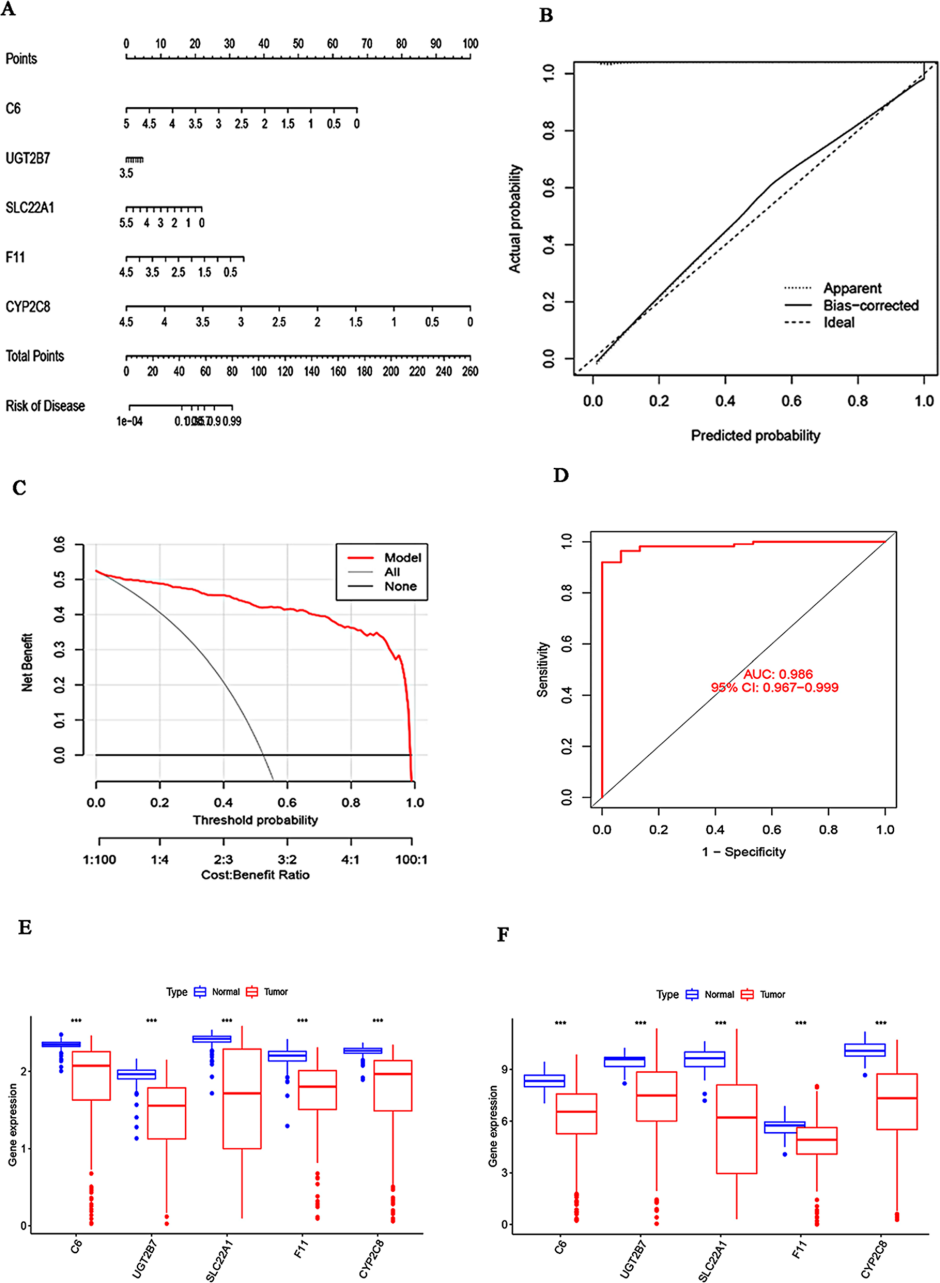
An RF model consisting of five genes was estimated. (**A**) A nomogram was developed to predict the risk of hepatocellular carcinoma (HCC) based on the RF model. (**B**, **C**) Calibration curve (**B**) and decision curve analysis (DCA) (**C**) were employed to assess the predictive probability of the nomogram model. (**D**) ROC analysis of the RF model was conducted using five-fold cross-validation on the TCGA dataset. (**E**, **F**) The differential expression of the five key genes between normal and HCC tissues was examined in the GSE25097 (**E**) and TCGA (**F**) datasets, respectively. RF, random forest

A nomogram was established to assess the risk of HCC and validate the diagnostic sensitivity of the RF model (Fig. 6A). The predictive efficiency of the nomogram model was evaluated using calibration and DCA. The calibration curve demonstrated a small error between the actual and predicted HCC probabilities, and DCA suggested that the nomogram may provide a basis for an accurate clinical diagnosis (Fig. 6B, C). Additionally, the RF model was validated using the TCGA-LIHC cohort, which included 50 normal and 374 tumor samples. The ROC curves showed a satisfactory AUC value of 0.986 (95% CI: 0.967–0.999), indicating that our diagnostic model was equally effective in the TCGA and GEO databases (Fig. 6D). Furthermore, the differential expression of the top five genes (*C6*, *UGT2B7*, *SLC22A1*, *F11*, and *CYP2C8*) in the RF model between normal and tumor tissues was validated using the GSE25097 and TCGA datasets. As shown in Fig. 6E and F, the expression trends of these five genes were consistent in both datasets, with all five genes being downregulated in HCC tumor tissues and showing significant p-values of less than 0.001.

Furthermore, we validated the differential expression of the top five genes (*C6*, *UGT2B7*, *SLC22A1*, *F11*, and *CYP2C8*) between normal and tumor tissues using the GSE25097 and TCGA datasets. Figure 6E and F show the consistent expression trends for these five genes across both datasets. Notably, all five genes were downregulated in HCC tumor tissues, with highly significant p-values (< 0.001) indicating their statistical significance.

### Estimation of the immune-infiltration feature in HCC and analysis of the immune cell correlation of core genes in RF models

**Fig. 7:**
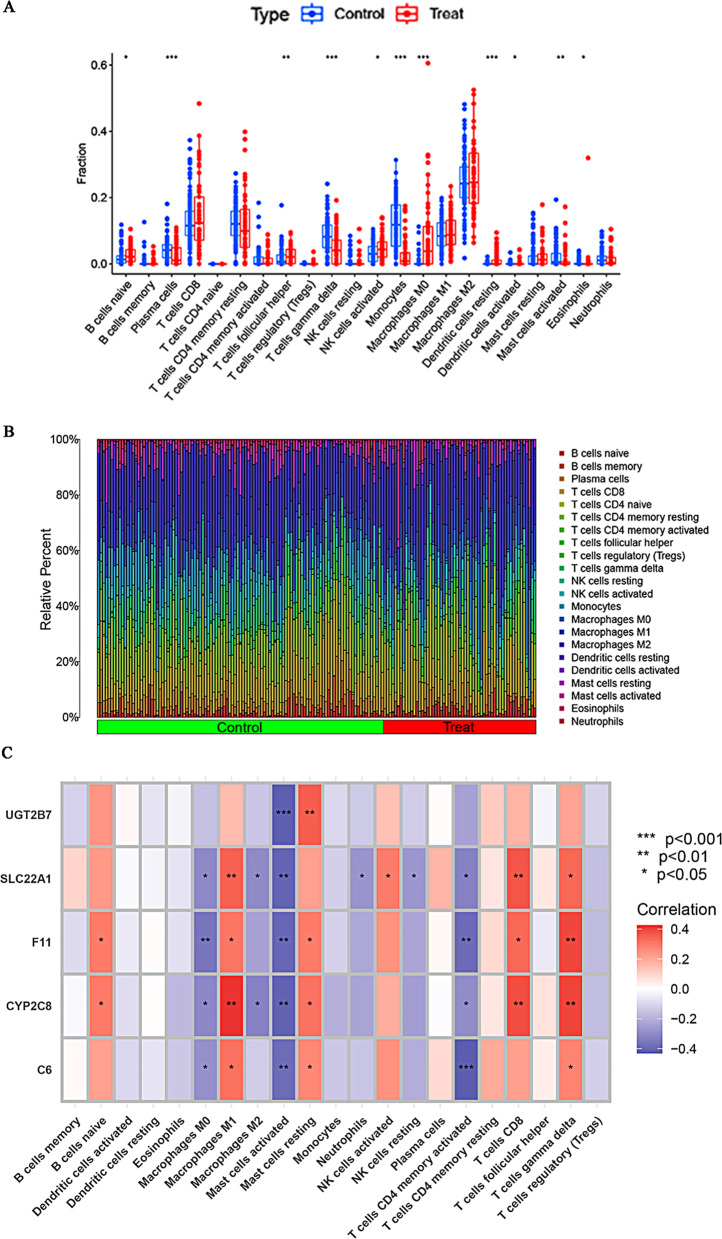
The immune-infiltration in hepatocellular carcinoma (HCC) and its correlation with core genes in RF models. (**A**) Boxplots were used to demonstrate the differential expression of 22 infiltrated immune cells between normal and HCC samples (*P < 0.05, **P < 0.01, ***P < 0.001). (**B**) The relative abundances of immune cells were compared between normal and HCC samples. (**C**) A heatmap was constructed to depict the association between the five genes in the RF model and the 22 infiltrated immune cells (*P < 0.05, **P < 0.01, ***P < 0.001). The colours blue and red indicated negative and positive correlations, respectively

The CIBERSORT algorithm was employed to evaluate the infiltration of immune cells in non-tumor and tumor tissues of the GSE25097 dataset. The findings revealed distinct expression of naïve B cells, plasma cells, follicular helper T cells, gamma delta T cells, activated NK cells, monocytes, M0 macrophages, resting dendritic cells, activated dendritic cells, activated mast cells, and eosinophils. Plasma cells, gamma delta T cells, monocytes, activated mast cells, and eosinophils were downregulated in HCC, whereas other cell types were upregulated (Fig. 7A, B). Subsequently, we investigated the correlation between the immune cells and core genes in the RF model. The results demonstrated a positive correlation between naïve B cells, M1 macrophages, resting mast cells, activated NK cells, CT8 + T cells, and T cell gamma delta, with the core genes in the RF model. Conversely, M0 macrophages, M2 macrophages, activated mast cells, neutrophils, resting NK cells, and activated CD4 memory T cells showed a negative correlation with core genes (Fig. 7C).

### FA-related gene prognostic model is an independent prognostic factor for patients with HCC

**Fig. 8:**
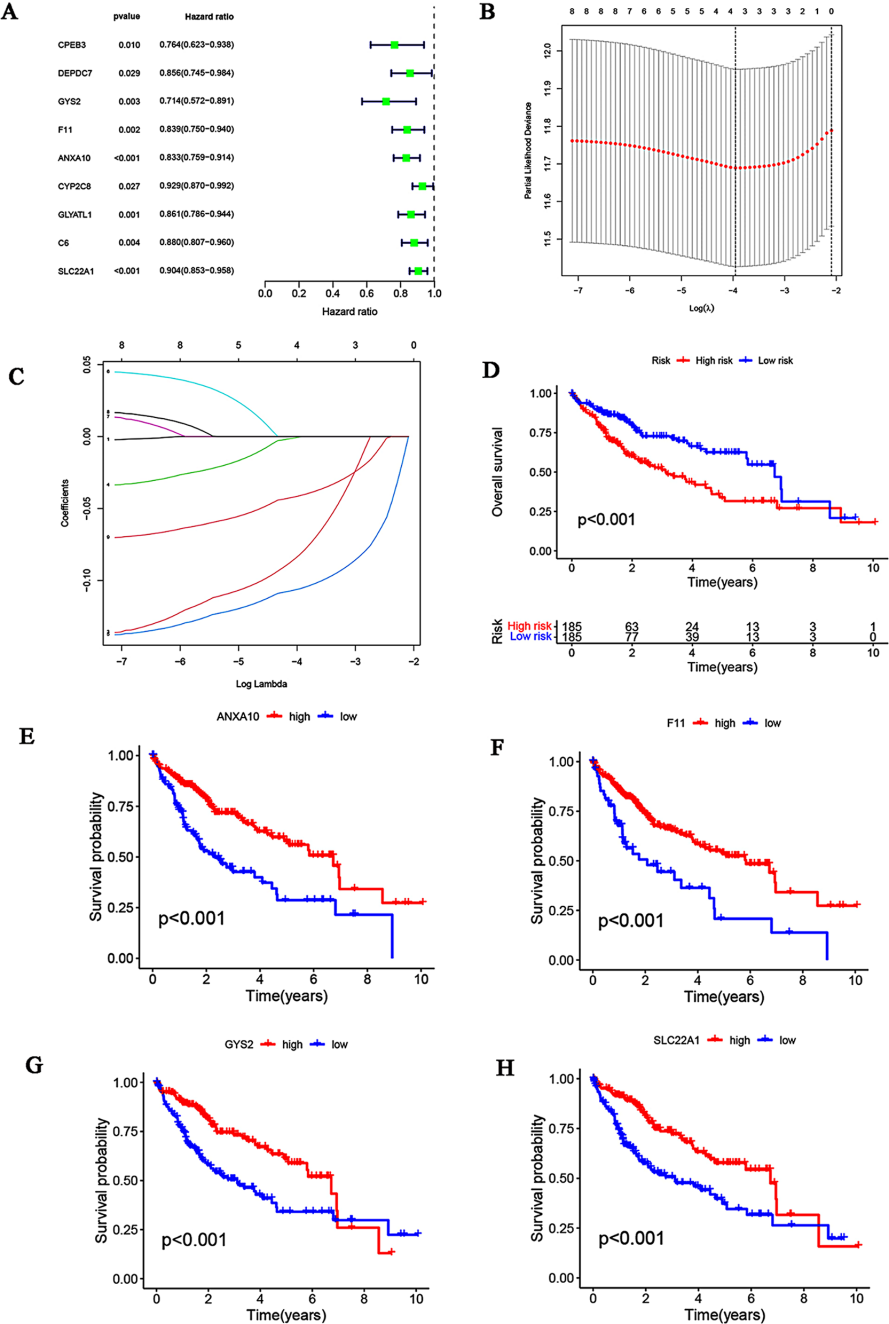
Construction of FA metabolism-related gene prognostic model in the TCGA cohort. (**A**) A forest plot was utilized to display the hazard ratio of nine FA-related genes with prognostic values, which were selected based on a univariate Cox regression analysis. (**B**, **C**) These nine genes with prognostic value underwent LASSO Cox regression analysis, resulting in the identification of four key FA-related genes (*CYS2*, *F11*, *ANXA10*, and SLC22A1) to build the predictive model for survival. (**D**) The OS rate of patients in high- and low-risk groups within the TCGA dataset was demonstrated utilizing Kaplan–Meier plots. (**E–H**) Separate Kaplan–Meier plots were generated for the survival analysis of each of the four model genes

Nine FA-related genes with prognostic value were identified among the 12 key overlapping genes using univariate Cox regression analysis (Fig. 8A). The FA-related prognostic signature was explored using LASSO Cox regression analysis (Fig. 8B, C), which was expressed using the following formula:

$$\eqalign{ Riskscore & = \left({ - 0.0821* ExpGYS2} \right) \cr & + \left({ - 0.000282* ExpF11} \right) \cr & + \left({ - 0.1057* ExpANXA10} \right) \cr & + \left({ - 0.04109* ExpSLC22A1} \right) \cr}$$

As shown in the Kaplan–Meier survival plot, patients with a high-risk score demonstrated a relatively lower OS rates than that of patients with a low-risk score (Fig. 8D). Similarly, a survival analysis of each model gene was conducted, and the results are shown in Fig. 8E and H. The results showed a relatively lower OS rate in patients with low expression of the four model genes than in patients with high expression.

**Fig. 9:**
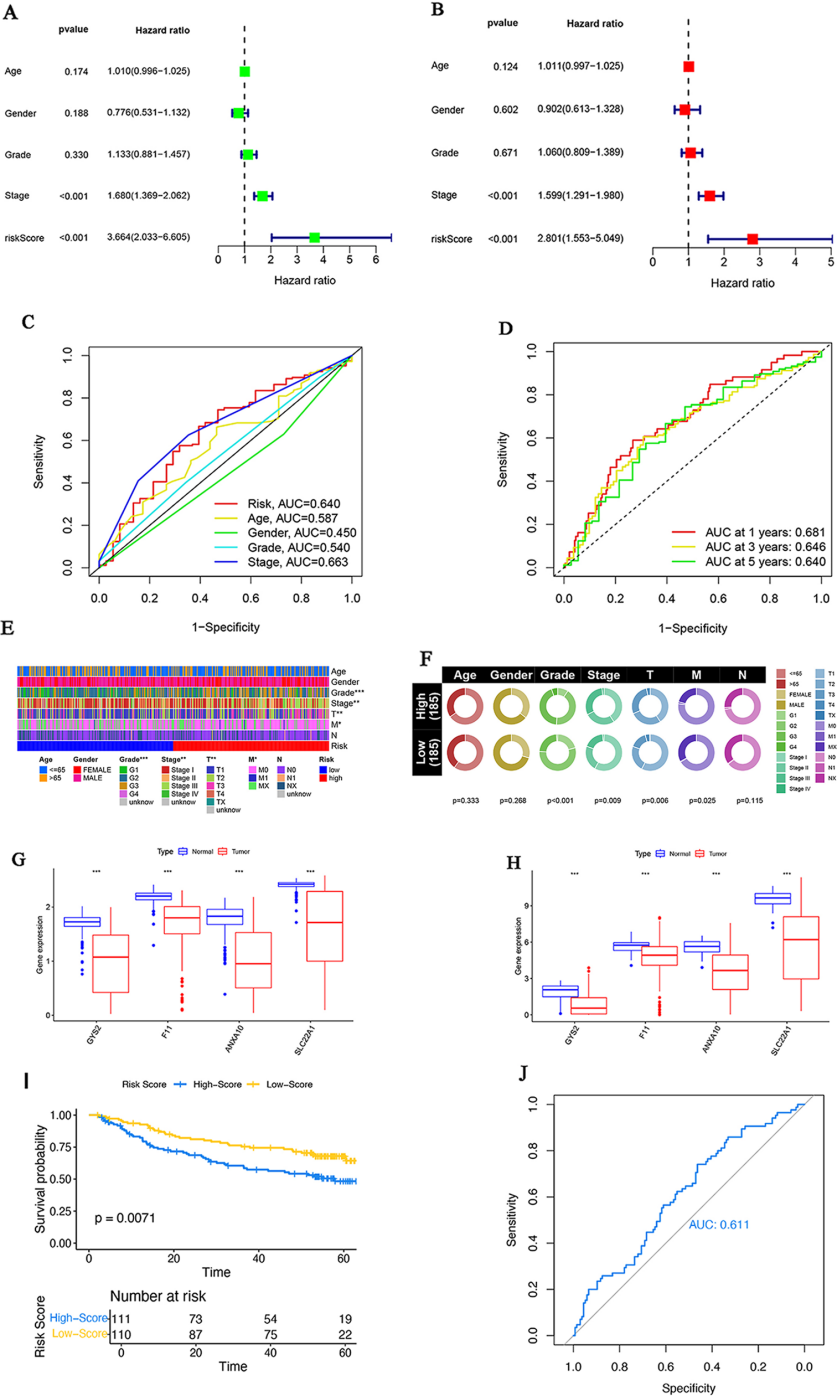
The exploration and evaluation of the independent prognostic value of our prognostic model. (**A**, **B**) The forest plots were used to demonstrate the hazard ratio of risk score and other clinical characteristics based on a univariate cox regression analysis (**A**) and multivariate cox regression analysis (**B**). (**C**) The prognostic prediction power of the constructed signature and clinical items was assessed based on the AUC of survival ROC curves. (**D**) The time-dependent AUC of the ROC curves was utilized to estimate the one-, three-, and five-year OS rates of patients from TCGA. (**E**, **F**) The clinical characteristic differences between high- and low-risk groups of patients within the TCGA dataset were visualized using a heatmap (**E**) and a circlemap (**F**) (*P < 0.05, **P < 0.01, ***P < 0.001). (**G**, **H**) The differential expression of four prognostic genes between normal and HCC tissues was examined in GSE25097 (**G**) and TCGA (**H**) datasets respectively. (**I**, **J**) The external validation of the prognostic model used GSE14520 dataset. The comparation of the overall survival rates between low and high risk-score groups (**I**). ROC curve was utilized to estimate the OS rates of patients (**J**)

Univariate and multivariate Cox regression analyses were performed to explore the independent prognostic value of the model. The results revealed a significant association between the risk score and OS in both univariate Cox regression analysis (hazard ratio [HR] = 3.664%, 95% confidence interval [95% CI] = 2.033–6.605, *P* < 0.001; Fig. 9A) and multivariate Cox regression analysis (HR = 2.801%, 95% CI = 1.553–5.049, *P* < 0.001; Fig. 9B), indicating that the risk score was an independent predictor for OS. Furthermore, to explore whether the risk score model was more sensitive in predicting OS than other clinical features, we conducted ROC analysis. The results showed that the AUC of the risk score model was greater than that of age, sex, and grade, and comparable to stage (Fig. 9C). Additionally, all AUCs for the one-, three-, and five-year OS rates of patients were > 0.5, indicating a high predictive value of the risk score for OS (Fig. 9D). Figure 9E and F show the correlation between the prognostic model and clinical characteristics, indicating significant differences in the clinical characteristics of grade, stage, T stage, and M stage between patients with a high-risk score and those with a low-risk score.

Moreover, we validated the differential expression of four model genes (*CYS2*, *F11*, *ANXA10*, and *SLC22A1*) between normal and tumor tissues in the GSE25097 and TCGA datasets. The results are shown in Fig. 9G and H. All five genes were downregulated in HCC tumor tissues, with *P* < 0.001.

Finally, the risk score model was validated in GSE14520 dataset, which also displayed a relatively lower OS rate in patients with low risk-score than in patients with high risk-score(*p* = 0.0071)(Fig. 9I). Meanwhile, the AUC for the OS rate of patients in the ROC analysis was 0.611, which greater than 0.5 (Fig. 9J). These external verifications further illustrated the reliability and validity of this model.

### The function of SLC22A1 in HCC

**Fig. 10:**
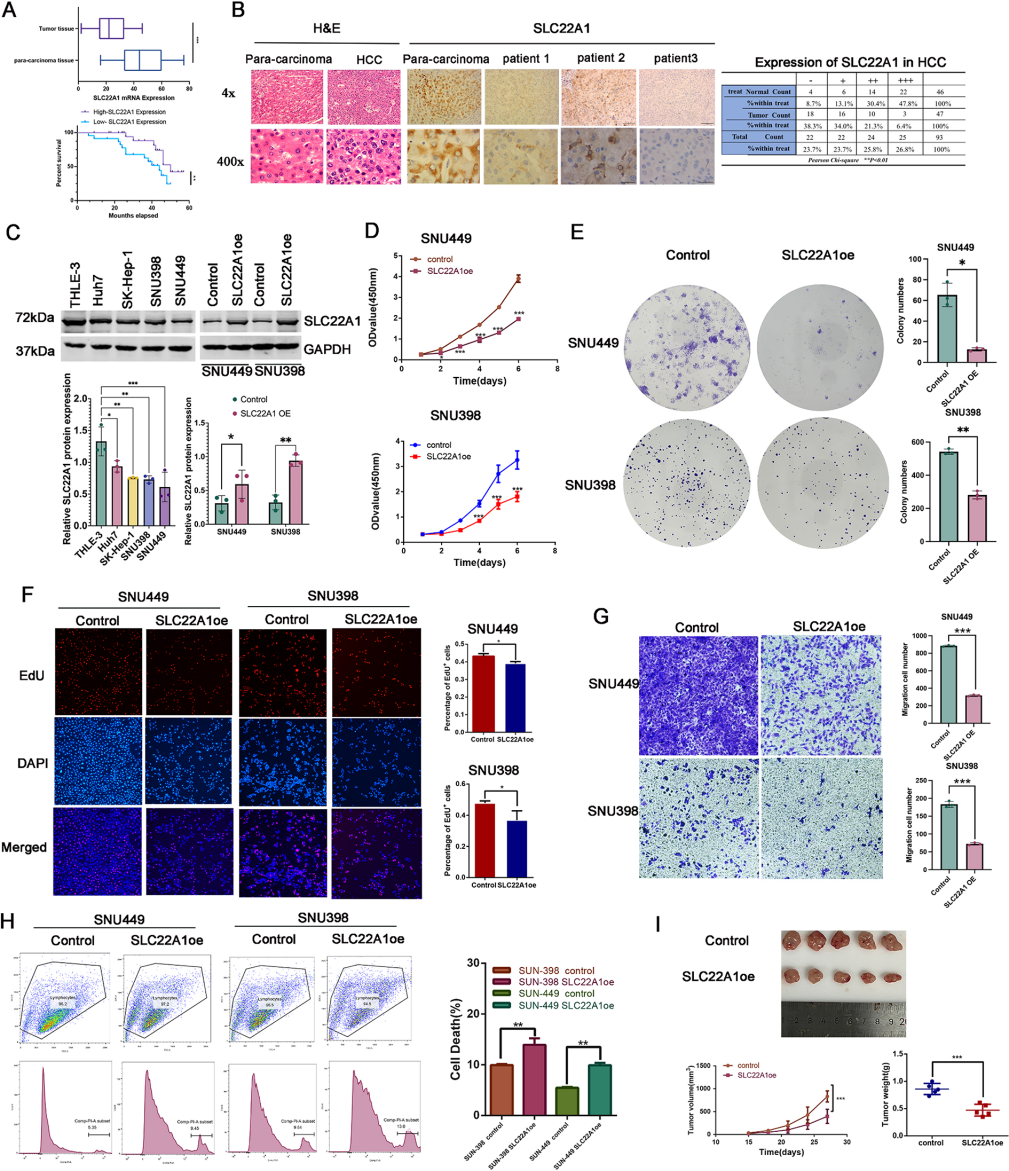
The role of SLC22A1 in determining the proliferation, migration, and death capacity of HCC cells. (**A**) The relative expression level of *SLC22A1* mRNA and the survivorship curve of 47 patients with HCC enrolled in this study were presented. (**B**) Representative IHC staining of H&E and SLC22A1 in para-carcinoma tissue and HCC tissues of patients with HCC. The SLC22A1 expression level was quantified using ImageJ (NIH, Bethesda, MD, USA) software. (**C**) The expression level of SLC22A1 in different HCC cell lines and SLC22A1 overexpression cells were confirmed by western blotting. (**D–F**) The impact of SLC22A1 overexpression on the proliferation of HCC cells was evaluated using CCK-8 assays (**D**), clone formation assays (**E**), and EDU assays (**F**). (**G**) The migratory ability of HCC cells was assessed using Transwell assays. (**H**) The effect of SLC22A1 overexpression on cell death was determined by flow cytometry. (**I**) The tumorigenicities of SUN449 cells were determined by subcutaneously injecting the cells (5 × 10^6^ cells/mouse; *n* = 5/group) into nude mice. Tumour volumes were measured using vernier calliper every 3 days for a duration of 27 days. The mice were sacrificed and tumour tissues were collected, and weighed at the end of the assay. HCC, hepatocellular cancer. Statistical significance was denoted by *P < 0.05, **P < 0.01, ***P < 0.001, and ns representing not significant

The *SLC22A1* gene, among the two key intersection genes (*SLC22A1* and *F11*), was chosen for further functional experimentation owing to its high weight coefficient of -0.04109 in riskscore formula. First, we verified the downregulation of SLC22A1 protein and mRNA levels in HCC tissues using IHC and qRT-PCR (Fig. 10A and B). Patients with low SLC22A1 expression exhibited lower survival rates (Fig. 10B). Western blotting analysis revealed the downregulation of SLC22A1 protein expression in four HCC cell lines (Huh7, SK-Hep-1, SNU449, and SNU398) compared with that in normal hepatocytes (THLE-3) (Fig. 10C).

SLC22A1 was overexpressed in SNU449 and SNU398 cells to study its function in HCC cells. Western blotting analysis confirmed the successful overexpression (Fig. 10C). SLC22A1 overexpression significantly reduced the proliferation ability of SNU449 and SNU398 cells, as demonstrated by CCK-8 and clone formation assays (Fig. 10D and E). EdU assay yielded similar results (Fig. 10F). Furthermore, transwell assays indicated that SNU449 and SNU398 cells faced more difficulty in metastasis after SLC22A1 overexpression than that in the wild-type cells (Fig. 10G). Additionally, SLC22A1 overexpression significantly increased cell death in SNU449 and SNU398 cells (Fig. 10H). In the in vivo assays, SLC22A1 overexpression resulted in a lower proliferative ability compared with that of the control groups (Fig. 10I). The findings indicate a suppressive effect of SLC22A1 overexpression on tumor progression.

## Discussion

We performed a systematic analysis to develop diagnostic and prognostic models using genes associated with FA metabolism in patients with HCC. Previous studies have used similar bioinformatics to develop potential biomarkers [29, 31]. Our study revealed substantial associations among FA-related gene signatures, and HCC diagnosis and prognosis. We identified 349 hub genes correlated with HCC and 125 hub genes correlated with FA clusters. Of these, 12 key genes (*CPEB3*, *ASPDH*, *DEPDC7*, *ETFDH*, *UGT2B7*, *GYS2*, *F11*, *ANXA10*, *CYP2C8*, *GLYATL1*, *C6*, and *SLC22A1*) were selected for diagnostic and prognostic analyses. Subsequently, five genes (*C6*, *UGT2B7*, *SLC22A1*, *F11*, and *CYP2C8*) were identified as potential novel diagnostic biomarkers and four genes (*CYS2*, *F11*, *ANXA10*, and *SLC22A1*) were identified as potential novel prognostic biomarkers in HCC. All seven model genes (*C6*, *UGT2B7*, *SLC22A1*, *F11*, *CYP2C8*, *CYS2* and *ANXA10*) were downregulated in HCC tissues compared with those in normal tissues. ROC analysis demonstrated the predictive value of both the diagnostic and prognostic models, as indicated by the AUC values.

C6, a complement protein and member of the membrane attack complex, interferes with the biological functions of HCC [32]. Our study revealed the downregulation of C6 and its diagnostic value in HCC, which is consistent with the results of other studies [33, 34]. UDP-glucuronosyltransferase 2B7 (UGT2B7) has been identified as a subtype classification biomarker for hepatocellular adenoma [35] and one as a gene signature that can accurately predict microvascular invasion in HCC [36], indicating its importance in liver cancer. Moreover, our research found consistent downregulation of UGT2B7 in hepatocellular adenoma [35]. Several studies have demonstrated the substantial function and downregulation of the organic cation transporter gene, *SLC22A1*, in HCC [37–39]. F11 has been identified as a signature with causal relationships with stroke and stroke subtypes [40], however, there is no relevant research on the relationship between F11 and HCC. The cytochrome P450 enzyme gene, *CYP2C8*, a member of the *CYP* gene family, participates in the proliferation, migration, invasion, and sorafenib resistance of HCC [41], indicating that CYP2C8 could be a potential target for the treatment of HCC. CYS2 gene, which encodes zinc finger protein with transcriptional activation properties, is involved in various cellular biochemical processes, development and stress response‌. However, there are no reports on the relationship between CYS2 and HCC have been found; therefore, further investigation is required. Annexin A10 (ANXA10), a calcium- and phospholipid-binding protein and a member of the annexin family, is one of the macrophage-related signatures in HCC [42]. Furthermore, a substantial reduction in the expression of ANXA10 in HCC [43], which is consistent with our findings.

Considering the important role of immunity in HCC, we evaluated the infiltration of immune cells into HCC and the correlation between RF model genes and immune cells. The results showed distinct differences in immune features between normal and HCC tissues as well as a strong relationship between the genes in the RF model and immune cells.

Finally, we conducted in vitro and animal experiments to verify the function of the selected key gene, *SLC22A1*, in HCC, further supporting the reliability of our study. Since SNU398 and SNU449 cell lines are often used in HCC related studies [44, 45], and the SLC22A1 expression of these two cells have been verified higher in SNU398 and lower in SNU449 which can represent the function of SLC22A1 at different expression levels, as well as the well cultured of these two cell lines in our laboratory, we finally choose these two cell lines to conduct further experiments. Despite the promising findings, our study is not without limitations. These include the possibility of sampling bias and the incomplete validation of the seven model genes’ functions, as well as the preliminary nature of the functional investigation of SLC22A1, which lacks substantial mechanistic understanding. To address these shortcomings and enhance our knowledge, more extensive and rigorous research is required.

## Conclusion

Our study systematically identified a signature of FA-related genes in patients with HCC, substantiated the considerable value of these genes in the diagnostic and prognostic evaluation of HCC, and certificated their pivotal contribution to the disease’s progression. These results have the potential to offer new strategies for the diagnosis, prognosis, and personalized treatment of HCC. Nevertheless, further research is necessary to confirm the effects of these signature genes in HCC.

## Electronic supplementary material

Below is the link to the electronic supplementary material.

Supplementary Material 1

## Data Availability

Sequence data that support the findings of this study have been deposited in the GEO (GSE25097 and GSE14520; http://www.ncbi.nlm.nih.gov/geo/) and TCGA (TCGA-LIHC; portal. gdc. cancer. gov) repositories.

## Abbreviations

AUC: Area under the curve
DCA: Decision curve analysis
DEG: Differentially expressed gene
FA: Fatty acid
GLM: Generalized linear model
HCC: Hepatocellular carcinoma
IHC: Immunohistochemistry
OS: Overall survival
RF: Random forest
ROC: Receiver operating characteristic
RT: Room temperature
SVM: Support vector machine
TOM: Topological overlap matrix
WGCNA: Weighted gene co-expression network analysis
XGB: Extreme gradient boosting
