# Supplementary figures and images for: Investigating the diagnostic and prognostic significance of genes related to fatty acid metabolism in hepatocellular carcinoma
Source: BMC Gastroenterol. 2024 Nov 15;24:409. doi: 10.1186/s12876-024-03495-2 (PMC11566841; doi:10.1186/s12876-024-03495-2)

Replicates 1

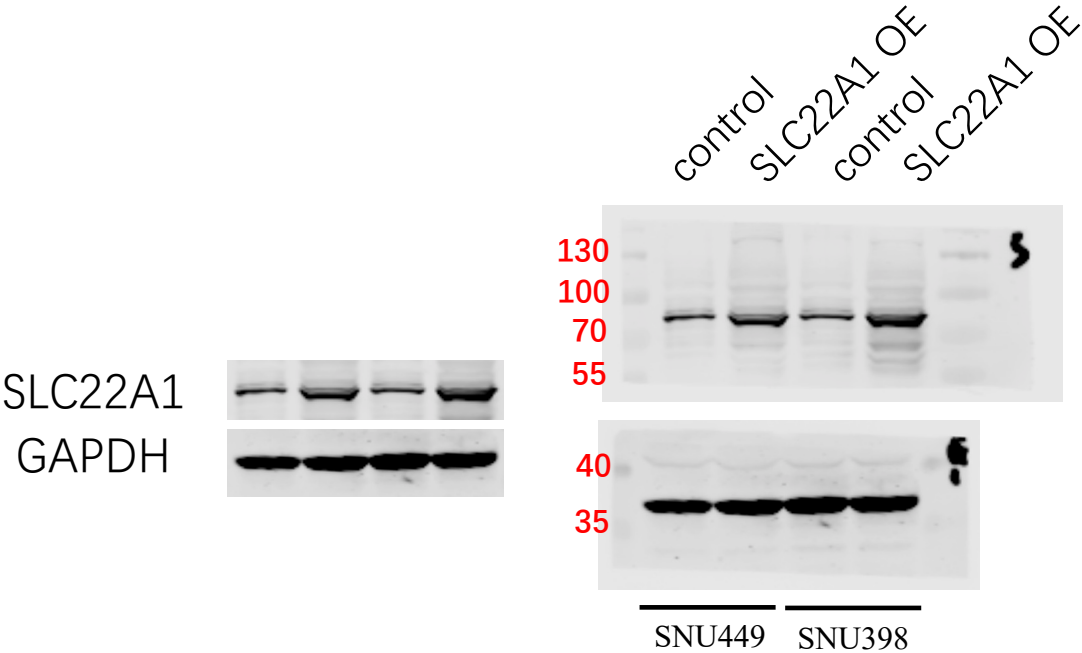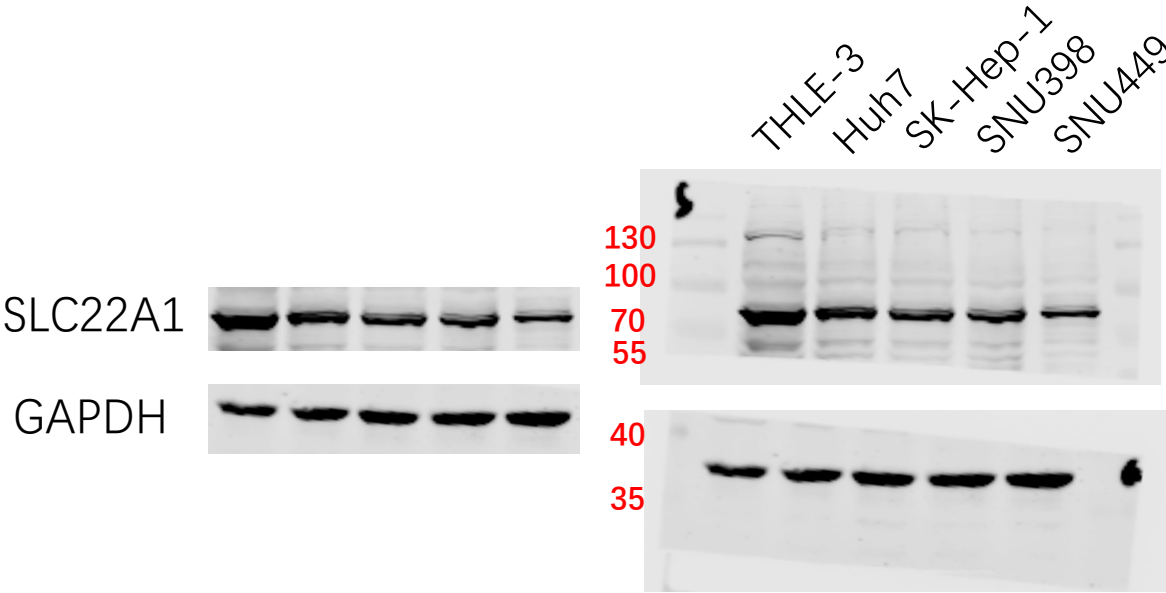

Replicates 2

SLC22A1

GAPDH

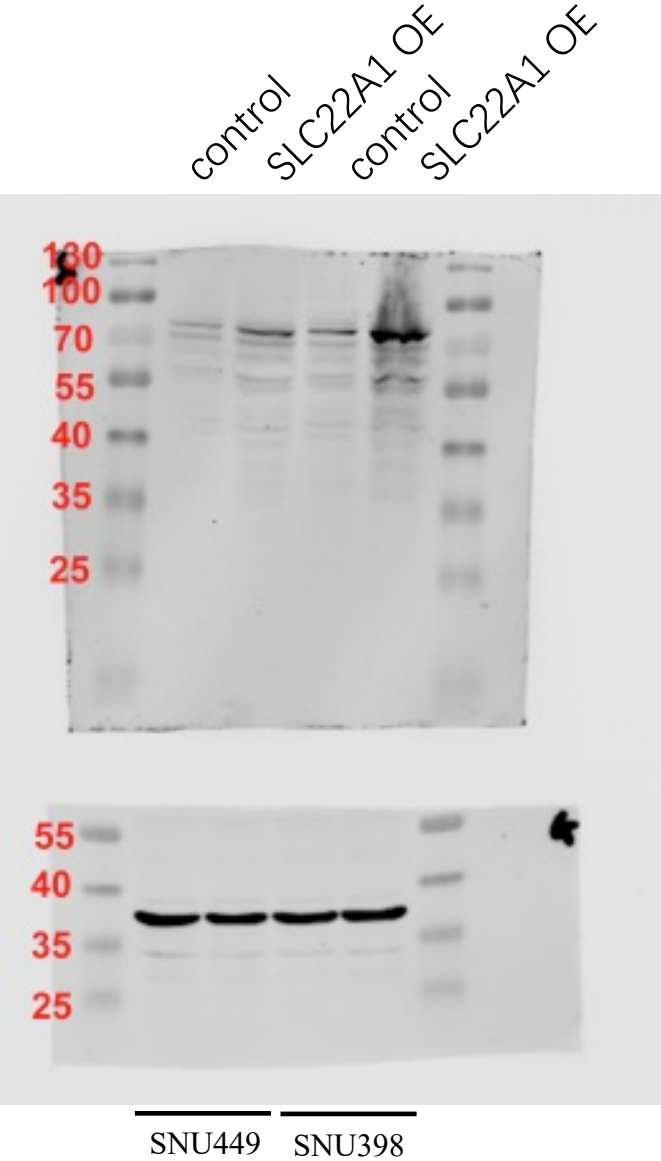

SLC22A1

GAPDH

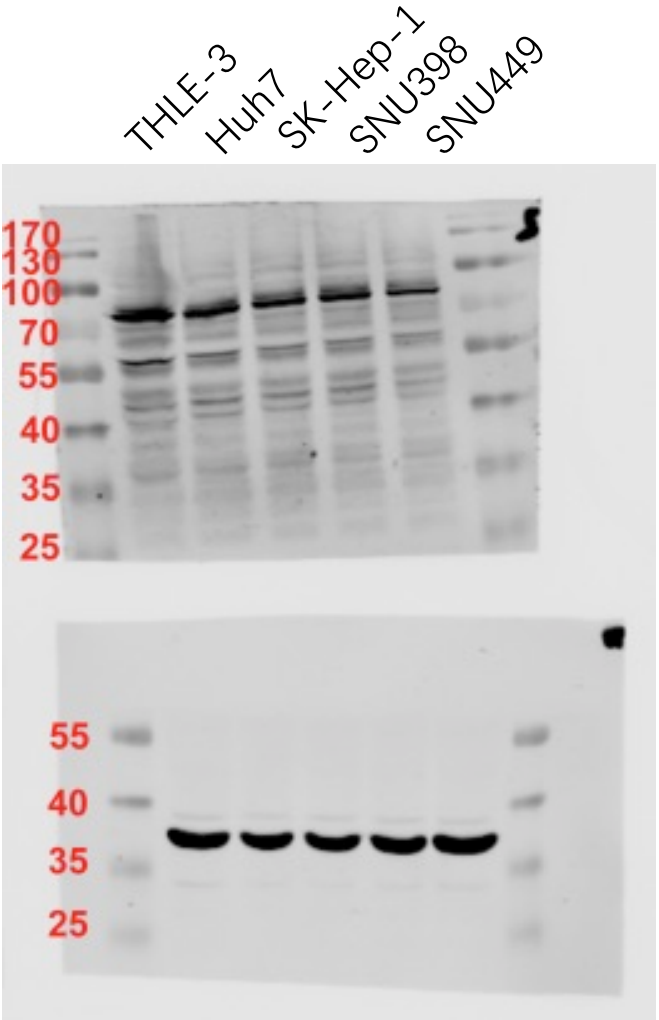

Replicates 3

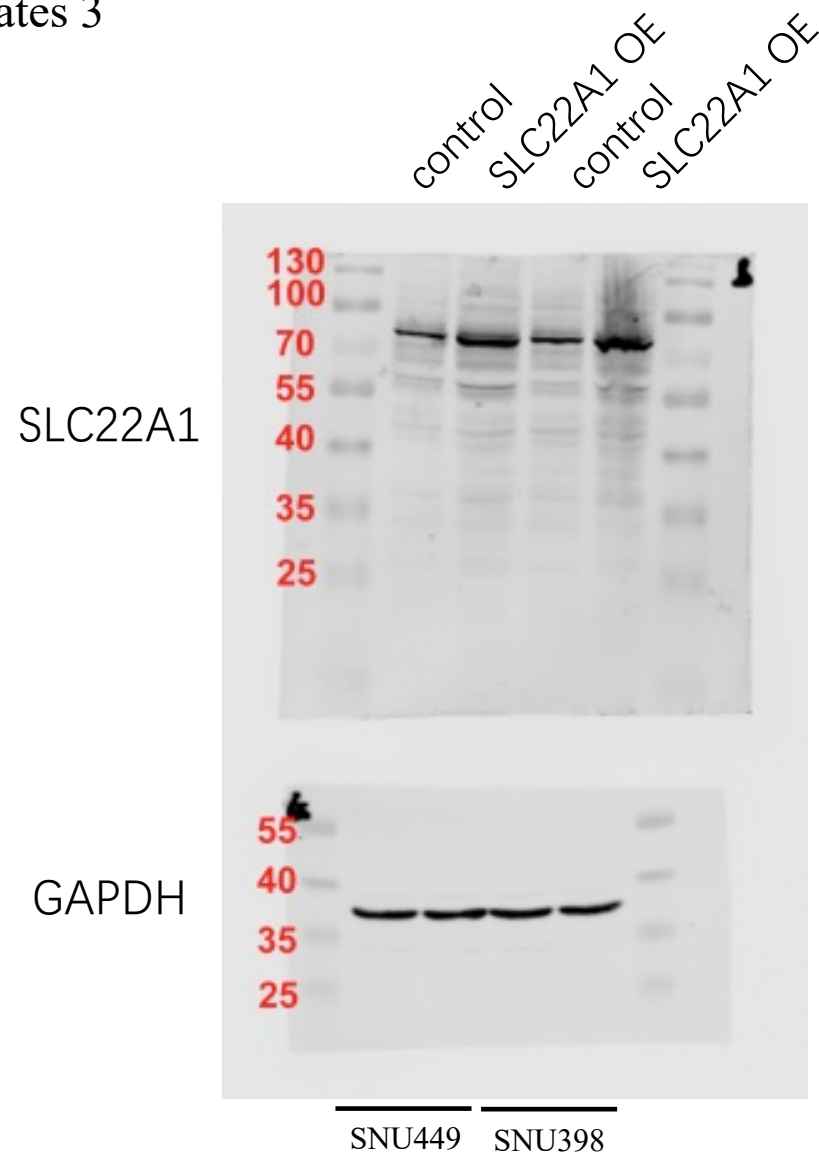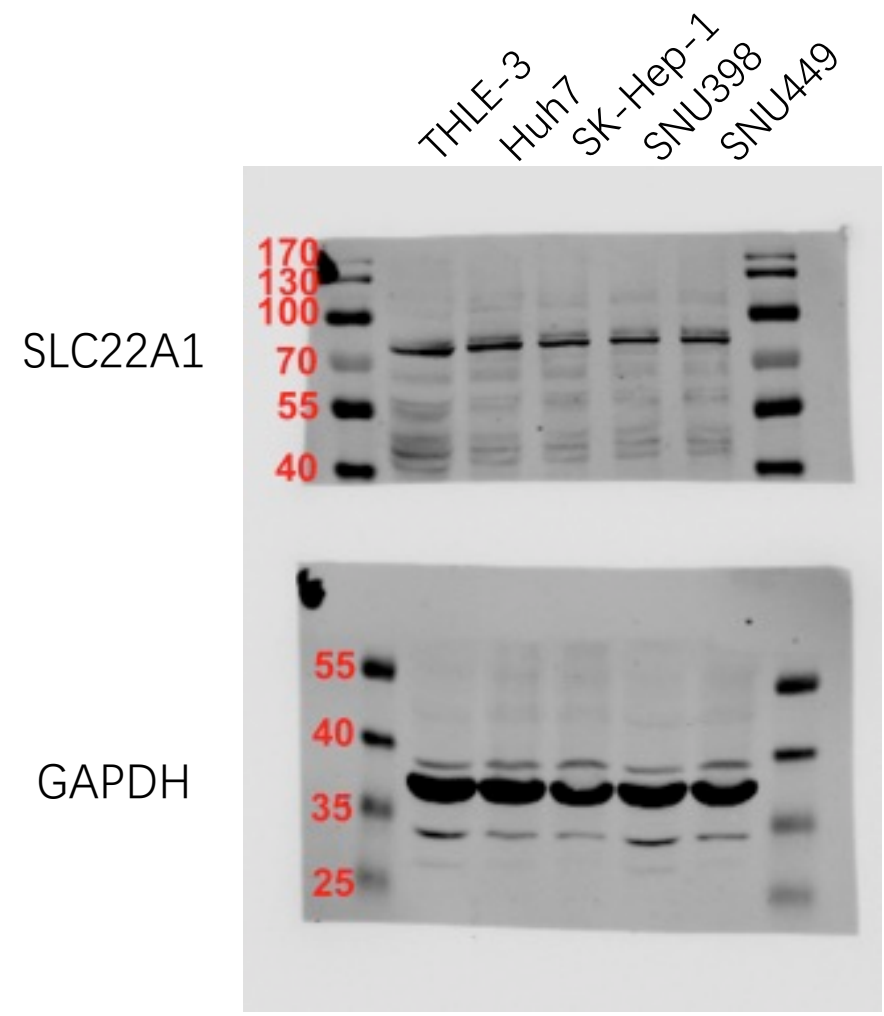

Supplement: Supplementary file 1 — Supplementary Material 1 [file 12876_2024_3495_MOESM1_ESM.pdf]
